# Traumatic Parafalcine Subdural Hematoma: A Case Report

**DOI:** 10.7759/cureus.60680

**Published:** 2024-05-20

**Authors:** Ami Changela, Robbie Buechler

**Affiliations:** 1 Internal Medicine, Edward Via College of Osteopathic Medicine, Charlotte, USA; 2 Neurology, Medical University of South Carolina, Lancaster, USA

**Keywords:** management of parafalcine hematoma, parafalcine subdural hematoma, traumatic fall, traumatic brain injury, interhemispheric hematoma, subdural hematoma

## Abstract

Parafalcine subdural hematoma is a rare subtype of intracranial hematoma. Brain hemorrhage, or hematomas, can occur in the brain or within the three layers that cover the brain. A subdural hematoma is trapped blood that develops between the inner layers and the tough outer covering called the dura. Typically, this is due to the tearing of the subdural or bridging veins. The patient in this report is an 85-year-old male who came to the emergency department following a fall on the second day with complaints of headache, neck pain, bilateral leg weakness, nausea, and vomiting. A computed tomography scan was performed in the emergency department, demonstrating an acute parafalcine subdural hematoma measuring 11 mm in thickness. This report will discuss the findings of interhemispheric hematomas and the rare parafalcine subtype and shed light on the diagnostic approach, medical and surgical treatment modalities, and prognosis.

## Introduction

According to the Centers for Disease Control and Prevention (CDC) Surveillance Report of 2019, approximately 1.5 million people in the United States suffer from traumatic brain injury (TBI) each year, accounting for over 60,000 deaths annually [1]. Subdural hematomas (SDHs) occur in 12%-29% of severe TBI cases and have mortality rates ranging from 40% to 60% [2]. Brain hemorrhage, or hematomas, can occur in the brain or within the three layers that cover the brain. An SDH is trapped blood that develops between the inner layers and the tough outer covering called the dura mater and arachnoid layer. Typically, this is due to the tearing of the subdural or bridging veins. While medical literature tends to focus on convexity SDHs, interhemispheric SDHs occur at the meningeal infoldings that encase the brain. We present a case report of a parafalcine SDH, which is a rare subtype of interhemispheric subdural hematoma (IHSDH).

IHSDH is an uncommon type of SDH due to its unusual location. It accounts for approximately 6% of all traumatic SDHs or 0.8% of all hospitalized patients after head trauma [3]. IHSDH occurs more commonly in older people over 60 years old. The male-to-female ratio is 2:1. The most common cause of this hematoma is trauma, especially over the occipital region, which accounts for 80% to 90% of cases [3]. Management of parafalcine hematomas was controversial until a recent study reported it to be a clinically benign finding [4]. As compared to patients with convexity SDH, patients with parafalcine SDH had a significantly lower incidence of radiographic progression and had no cases of neurologic deterioration, neurosurgical intervention, or mortality (all P<0.005) [4]. According to a retrospective cohort study, patients with parafalcine SDHs who have an initial Glasgow Coma Scale (GCS) score of <15, who are 60 years of age or older, and who have other TBIs on their CT scan are at increased risk of complications and require hospitalization [2]. Patients without these findings are at low risk and may not need hospitalization [2]. However, rare complications of parafalcine hematoma can include falx syndrome, contralateral monoparesis of the lower extremity, or contralateral hemiparesis with lower limb weakness predominating, which may require neurosurgical intervention [5].

This case reports a classic example of parafalcine SDH, highlighting the mechanism of injury, symptoms, imaging findings, and treatment course during the hospital stay. The patient’s only symptoms were headache, neck pain, bilateral lower extremity weakness, nausea, and vomiting, with minimal neurological deficits and no altered consciousness post-occipital head injury. This case is educationally important for both specialist and non-specialist doctors who evaluate TBI patients but are not familiar with the radiological findings of interhemispheric or parafalcine SDHs.

## Case presentation

An 85-year-old male with a past medical history of hypertension, a cochlear implant, and prostate cancer presented to the emergency department for a fall that occurred two days prior. The patient stated that he was climbing over a banister on his porch, where he tripped, fell, and hit the back of his head. He denied syncopal events and dizziness before the fall. The patient did not experience a loss of consciousness. He stated that he has had a headache since the fall at the base of his head. His only initial complaint was neck pain, for which he took ibuprofen. He did report an episode of nausea and vomiting at home. He later developed bilateral leg weakness. He denied visual disturbance, dizziness, or aphasia. He denied the current use of blood thinners. The wife, who was present at the bedside, stated that the patient is at his baseline and has been behaving normally.

His physical exam findings included an elevated blood pressure of 165/99, a temperature of 97.7 °F, a heart rate of 72, a respiratory rate of 20, and 96% oxygen saturation. The general medical exam was unremarkable. Neurology and neurosurgery were consulted for an in-depth neurological evaluation. The patient was alert and oriented to person, place, and time with normal speech. Sensations to light touch were intact bilaterally; there was a mild motor deficit noted in the lower extremity with muscle strength of 4/5. Cranial nerves and cerebellar function were intact. The patient was given Zofran and morphine in the emergency department for symptomatic relief.

**Figure 1:**
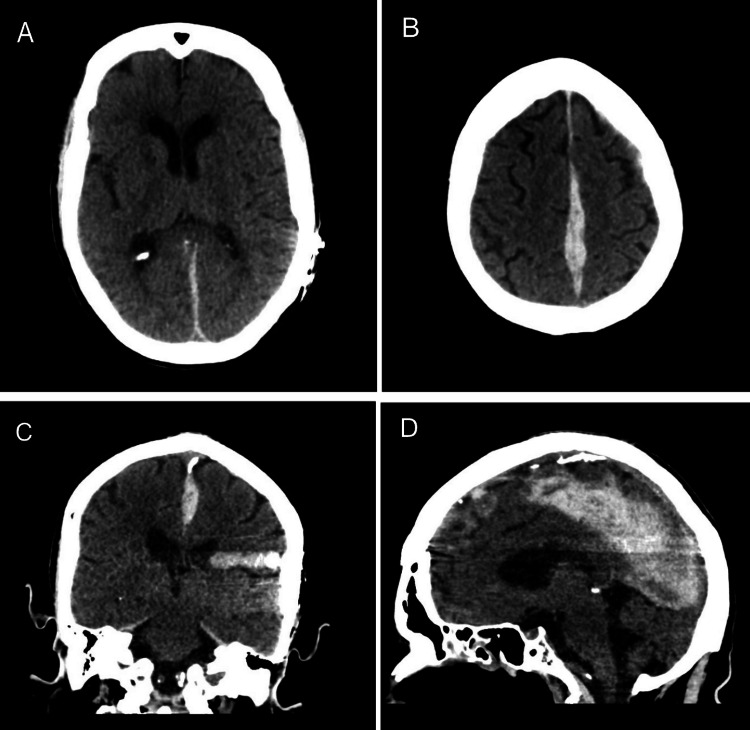
Non-contrast CT scan of the head. Panels A and B show the axial views of a hyperattenuating parafalcine subdural hematoma measuring up to 11 mm in thickness. Panel C shows a coronal view of the hematoma in the location of the falx cerebri, with some mild artifacts on the right, secondary to the patient's cochlear implant. Panel D shows a sagittal view of the parafalcine subdural hematoma extending into the falx cerebelli.

Non-contrast computed tomography (CT) of the head was performed, which resulted in findings of a parafalcine SDH measuring up to 11 mm in thickness, chronic intracranial parenchymal volume loss, and periventricular microangiopathy (Figure 1). A cervical spine CT without contrast revealed no fractures in the cervical region. Following consultation with a neurosurgeon, it was advised to conduct another non-contrast CT scan of the head after six hours. The subsequent CT scan showed no additional hematoma developments, and the neurosurgical team determined that no surgery was necessary. The patient, with a GCS score of 15 and stable hemodynamics, was deemed fit for discharge with outpatient monitoring by the neurosurgery team.

However, upon leaving the hospital, the patient experienced a near syncopal episode, nausea, pallor, and difficulty walking. Neurosurgery recommended a third non-contrast CT scan of the head after six hours, overnight observation, and an evaluation for syncope. By the following day, the patient's symptoms had resolved entirely, and the subsequent CT scan showed no further enlargement of the SDH. After a thorough syncope evaluation and an unchanged third CT scan, cardiology, neurology, and neurosurgery approved the patient's discharge. Within a week of discharge, the patient visited neurosurgery for a follow-up, reporting no residual symptoms such as headaches, vision changes, weakness, numbness, or syncopal episodes. The patient achieved complete recovery without any complications or leg weakness impairment.

## Discussion

This case presentation will raise awareness within the medical community regarding the identity of parafalcine SDH, imaging study results, management, and favorable prognostic outcomes. While there are increasing reports of IHSDHs [6], clear guidelines for managing such cases are lacking [7]. Given the controversial nature of IHSDH treatment and management, the approach is tailored to the patient’s neurological findings related to hematoma severity. According to the prognostic analysis by Wang et al., patients with a low GCS score and greater interhemispheric hematoma thickness tend to have poorer outcomes [8]. Numerous studies suggest considering surgery if clinical signs and symptoms progress, such as worsening lower extremity weakness, bilateral lower extremity paralysis, persistent increased intracranial pressure (>30 mmHg), or hematoma thickness exceeding 15 mm [8-10].

In our case, the patient exhibited minimal neurological weakness and no paralysis or loss of consciousness following a traumatic fall. Although the patient experienced headaches, they resolved with appropriate management of blood pressure and pain medications during hospitalization. Two non-contrast head CT scans showed hematoma thickness consistent with 11-11.6 mm. However, at discharge, the patient experienced a near syncopal episode with some nausea. A repeat head CT revealed no new acute changes. Aspirin was discontinued, and strict systolic BP <160 was maintained with antihypertensive medications. The patient recovered well with conservative medical management and did not require further surgical intervention.

This case report aims to educate healthcare professionals about a rare form of IHSDH, particularly parafalcine SDH. Prompt assessment of clinical and neurological conditions, along with imaging and emergent care, is crucial for identifying treatment strategies and achieving better patient outcomes in parafalcine SDHs.

## Conclusions

This case report highlights the rare subtype of SDH known as parafalcine SDH. It intends to educate medical students, doctors, and specialists regarding its common symptoms yet unique imaging findings. Our patient had an excellent prognosis and achieved a full recovery.
